# Frequency of latent tuberculosis in patients receiving Anti-TNF-Alpha therapy

**DOI:** 10.4314/ahs.v23i2.14

**Published:** 2023-06

**Authors:** İpek Coşkunol, Onur Turan, Aysegul Baysak, Dilek Solmaz, Gerçek Can

**Affiliations:** 1 Izmir Atatürk Research and Training Hospital, Chest Diseases Department, İzmir-Turkey; 2 Izmir Katip Celebi University, Chest Diseases Department, izmir-Turkey; 3 Çamlica Medipol University Hospital, Chest Diseases Department, İstanbul -Turkey; 4 Izmir Katip Celebi University, Rheumatology Department, İzmir -Turkey; 5 Dokuz Eylül University, Rheumatology Department, İzmir-Turkey

**Keywords:** Anti-TNF-alpha, tuberculosis, tuberculin skin test

## Abstract

**Setting-Objective:**

In this study, it was aimed to reveal the incidence of tuberculosis development in patients receiving tumor necrosis factor-alpha (TNF-α) blocker therapy, despite tuberculosis chemoprophylaxis.

**Design:**

520 patients who were receiving anti TNF-α treatment in the last 3 years were evaluated retrospectively. Radiological imaging tuberculin skin test (TST), history of tuberculosis, BCG vaccine, chemoprophylaxis administration, used anti TNF-α drugs were recorded.

**Results:**

There were 265(51.0%) of the patients with ankylosing spondylitis (AS), 175(33.7%) with rheumatoid arthritis, 35(6.7%) with Crohn's, 10(1.9%) with ulcerative colitis (UC), 21(4.0%) with psoriatic arthritis, 14(2.7%) with psoriasis vulgaris. In total, 455 (79.6%) patients were given INH prophylaxis. Active tuberculosis development was observed in five patients (4: pulmonary,1: extrapulmonary; 3: UC, 2:AS) who all received anti TNF-α treatment (0.96%), infliximab. Three patients had tuberculosis disease in the 6th month, and the other 2 patients in the 5th and 24th month of their anti TNF-α treatments, and two had 9-month, and 1 had 6-month chemoprophylaxis history.

**Conclusion:**

The incidence of tuberculosis development in patients treated with anti TNF-α was found to be higher than the general population. In our country, where tuberculosis is still prevalent, patients receiving Anti TNF-α treatment (especially infliximab) should be carefully questioned and examined about tuberculosis.

## Introduction

Tumor necrosis factor- alpha (TNF-α) is a broad-spectrum hormone produced by monocyte and macrophage, in polypeptide structure and responsible for many biological activities in the target cell 1. TNF-α has a role in many events such as immunity, inflammation, tissue repair and infection 2. When TNF-α is inhibited, the immune system is adversely affected and the development of serious infections becomes easier 3. Since TNF-α also plays a role in the development of autoimmune diseases, it is associated with the developmental pathophysiology of diseases such as rheumatoid arthritis (RA), ankylosing spondylitis (AS), and Crohn's disease. Hence, TNF-α antagonists are used as an effective treatment of such diseases, especially collagen tissue diseases 3, 4.

TNF-α has a strategic role in the immune system response developed against Mycobacterium tuberculosis; increased antibacterial activity of macrophages causes lymphocyte migration and proliferation to the inflammation region through the release of cytokines and chemokines 5. This mechanism contributes to formation of granulomas and prevents bacilli from reproducing and spreading through confinement 4, 6. Because of this reason, the decrease in TNF activity suppresses the formation of the granulomatous inflammation process 6. Anti-TNF drugs can facilitate development of tuberculosis disease by disrupting this mechanism.

Tuberculosis, which is a treatable disease, infects approximately 1/3 (2.3 billion) of the world population. About 8 million people get sick every year from this infection pool and 1.6 million people die 7. It is thought that there is about 15-25% latent tuberculosis infection in the adult population in Turkey 8. However, due to mandatory BCG vaccination, it is difficult to determine an accurate prevalence.

The risk of tuberculosis during anti-TNF therapy gains a great importance, especially in communities with a high prevalence of tuberculosis. Rules for the use of anti-TNF-α agents, in order to screen patients for latent tuberculosis during treatment and to give tuberculosis preventive treatment to patients at risk, to be based on some principles; various guides have been published 9.

In this study, the follow-up and the frequency of tuberculosis development of 196 patients who received anti-TNF therapy were included.

## Material and Method

There were 520 patients who were receiving anti TNF-α treatment and admitted to our chest diseases outpatient clinic in the last 3 years were evaluated retrospectively in terms of the frequency of tuberculosis development. INH prophylaxis for tuberculosis was given to our patients 1 month before starting anti-TNF treatment.

Patients diagnosed with rheumatoid arthritis (RA), ankylosing spondylitis (AS), rheumatoid arthritis (RA), Crohn's, ulcerative colitis, psoriatic arthritis (PSA) and psoriasis vulgaris were included in the study. These patients were evaluated according to the guidelines on anti-TNF use and the safe anti-TNF instruction manual 9,10.

Hospital registration systems, polyclinic and service anamnesis and examinations of all patients were examined in detail. Radiological imaging of the patients, tuberculin skin test (TST) results, physical examination findings, tuberculosis history, BCG vaccine, chemoprophylaxis status and duration, which group received anti TNF-α drug, and how long they used it were recorded. The results of acid-fast bacilli (ARB) in sputum sent from patients with suspected tuberculosis were analysed.

In addition, the type of the disease for which anti-TNF therapy was given, information about the immunosuppressive treatments applied for this disease and the data about the selected anti-TNF-α agent in the patients included in the study and whether the patient's received chemoprophylaxis or not were recorded.

### Ethics approval

Ethics committee approval for the study protocol was obtained from the Ethics Committee of the İzmir Katip Çelebi University in accordance with guidelines of the Declaration of Helsinki, and permission for the study was obtained from the Ministry of Health of the Republic of Turkey. The requirement for informed consents was waived due to the retrospective design of the study.

## Results

**Figure 1:**
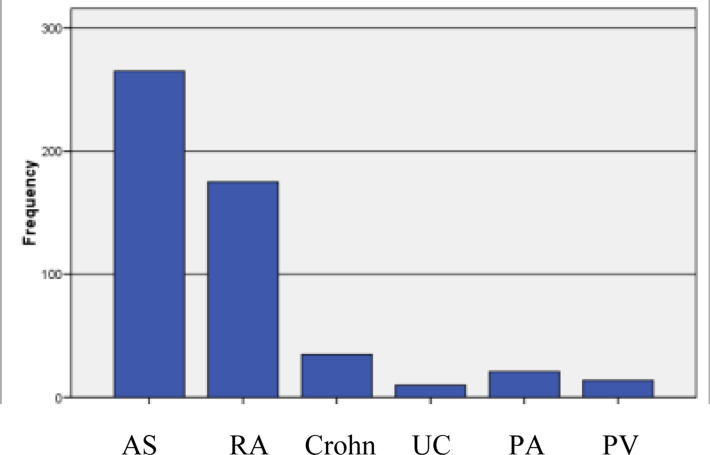
The diagnosis of patients receiving anti-TNF therapy Ankylosing spondylitis: AS, rheumatoid arthritis: RA, ulcerative colitis: UC, psoriatic arthritis: PA, psoriasis vulgaris: PV

Of 520 patients, 286 (55%) were male, 234 (45%) were female, and the mean age was 43.4 ± 12.6. Also, 265 (51.0%) of the patients were receiving anti TNF therapy due to the diagnosis of ankylosing spondylitis, 175 (33.7%) due to rheumatoid arthritis, 35 (6.7%) due to Crohn's disease, 10 (1.9%) due to ulcerative colitis, 21 (4.0%) due to psoriatic arthritis and 14 (2.7%) due to psoriasis vulgaris (figure 1). According to the results of TST; the number of patients measured 0 mm was 117 (23.2%), the number of patients between 1 and 4 mm was 57 (11.3%), and 5 mm and above was 331 (65.5%). In total, 455 (79.6%) patients were given INH prophylaxis. Active tuberculosis was observed in five patients who received anti TNF-α treatment (0.96%). Three of the patients were diagnosed with ulcerative colitis, two were diagnosed with ankylosing spondylitis, and 5 were receiving infliximab treatment. Pulmonary tuberculosis developed in 4 patients and extrapulmonary tuberculosis developed in 1 patient. 3 patients had tuberculosis disease in the 6th month and the other 2 patients in the 5th and 24^th^ month of their Anti TNF-α treatment. Two of the patients who developed tuberculosis had received chemoprophylaxis treatment for 9 months, and 1 received for 6 months.

12 of our patients had a history of tuberculosis diagnosis and treatment prior to anti TNF -α alpha therapy. It was observed that 505 cases had a tuberculin skin test during the admission. According to the results of TST; the number of patients measured 0 mm was 117 (23.2%), the number of patients between 1 and 4 mm was 57 (11.3%), and 5 mm and above was 331 (65.5%). Quantiferon test (epicentre) was applied in 7 patients whose TST results were suspicious, 2 patients had positive and 5 patients had negative results.

15 of the patients who underwent radiological imaging had sequelae changes in the lung, 12 patients had pulmonary involvement findings of rheumatological disease (or systemic disease with anti-TNF), 6 patients had lung nodules, and 2 patients had bronchiectasis.

In total, 455 (79.6%) patients received INH prophylaxis of 5 mg / kg / day (maximum 300 mg / kg), 92.5% of the patients who received prophylaxis received preventive treatment for 9 months, 4.4% for 6 months, 2.6% for 12 months, and 0.5% for less than 6 months. 331 (73.6%) of the patients who were given chemoprophylaxis had TST values of 5 mm or greater, 109 (24%) of them were found to have 0 mm TST, since the test result is anergic when repeated within 1 week, and 15 (2.4%) are in the group with high risk of tuberculosis, such as tuberculosis sequelae in the lung film, they had received treatment for latent tuberculosis infection.

Active tuberculosis development was observed in five patients who received anti TNF-α treatment (0.96%). Three of the patients were diagnosed with ulcerative colitis, two were diagnosed with ankylosing spondylitis, and 5 were receiving infliximab treatment. Pulmonary tuberculosis developed in 4 patients and extrapulmonary tuberculosis developed in 1 patient. 3 patients had tuberculosis disease in the 6th month and the other 2 patients in the 5th and 24th month of their Anti TNF-α treatment. Two of the patients who developed tuberculosis had received chemoprophylaxis treatment for 9 months, and 1 received for 6 months.

## Discussion

The principle objective of this study is to reveal the prevalence of tuberculosis in patients receiving anti TNF-α therapy and to draw attention to the risk of developing tuberculosis as one of the significant side effects of these frequently used drugs.

Biological agents such as adalimumab, infliximab and etanercept that antagonize the activity of TNF-α, an important cytokine in chronic inflammatory diseases are widely used as a powerful treatment option for many rheumatological diseases in our country 11, 12. However, TNF-α is also a cytokine that plays a role in the formation of granulomas that prevents the spread of bacilli by 4. Studies have shown that active tuberculosis may develop in these individuals shortly after receiving anti-TNF-α treatment 13. Therefore, increased risk of developing tuberculosis with this frequently used treatment option poses a significant problem especially in a country with a high prevalence of tuberculosis like our country 14, 15.

It is stated that the use of anti TNF in our country increases the risk of developing tuberculosis 10-20 times 9. In a study conducted in Turkey, 3 patients were diagnosed with tuberculosis in the 3-year follow-up of 192 patients who were receiving anti-TNF therapy for rheumatologic disease; active tuberculosis development rate in this study is 1.5% 16. In a study published by Cagatay et al. in 2009, this number was 6 cases of tuberculosis in 702 patients, and active tuberculosis development rate was 0.85% 17. A Turkish study reported that tuberculosis developed in 1.16% of 1887 patients who were receiving TNF-α antagonists 18. The incidence of tuberculosis development in rheumatology patients receiving anti TNF alpha therapy in a review published in 2020 by Sartori et al. was found to be 9.62 (0.96%) in 1000 patients 19. In these studies, the rates of tuberculosis development in patients receiving anti-TNF therapy were found to be similar to those in our study.

According to the data of the Ministry of Health in our country, the incidence of tuberculosis is 14.6 per hundred thousand in 2017 20. Tuberculosis developed in five (0.97%) of the 520 patients included in our study during anti TNF-alpha treatment. Accordingly, the rate of tuberculosis development is approximately 66 times higher and is considerably higher than the incidence of tuberculosis in our country.

Many studies have demonstrated that tuberculosis develops frequently after infliximab treatment 13. In a meta-analysis, the risk of developing tuberculosis in rheumatoid arthritis patients using infliximab increased 4 times. The incidence of tuberculosis in patients receiving infliximab was reported to be 0.70% 21. In our study, development of tuberculosis in patients using only infliximab suggests that care should be taken in terms of tuberculosis, especially in the patient group given this biological agent.

The presence of latent tuberculosis infection (LTE) should be investigated before using anti-TNF-α therapy, it is necessary to decide whether there is a need for prophylaxis. The most practical and cost-effective test used in this regard is TST. However, false negativity rate is high in TST responses in immunosuppressive patients and this situation may reflect the proportion of the patient group that needs to be given chemoprophylaxis less. In the recent years, Interferon Gamma Release Assay (IGRA) tests that measure the interferon gamma response against tuberculosis bacillus can also be used in the diagnosis of LTE in patients who have been receiving anti-TNF therapy. Quantiferon TB-Gold is a test used to measure the IFN-γ level in the blood, which is formed in response to some antigens specific to Mycobacterium tuberculosis, by ELISA method 22. However, since its high cost, the general tendency in our country is to apply this test in confusing and suspicious TST results. The use of Quantiferon test in only 7 patients in our study may be due to the fact that it is not cost effective and this test cannot be performed in every centre.

Another factor that plays a role in the decision of LTBI treatment before anti TNF therapy is the presence of suspicious radiological findings. Guidelines also express the significance of detailed tuberculosis exposure history, as well as TST, radiological imaging of the lung in terms of tuberculosis prophylaxis 23. The presence of lesions that may be sequelae of tuberculosis in lung imaging played a role in the choice of starting chemoprophylaxis in 2.9% of the patients in our study.

The retrospective design of the study may be a limitation. A prospective study planning whether tuberculosis will develop in patients receiving anti-TNF will provide more accurate and objective results.

## Conclusion

The incidence of tuberculosis development in patients treated with Anti TNF was found to be higher than the general population. In Turkey, where tuberculosis is still prevalent, patients receiving Anti TNF-a treatment (especially infliximab) should be carefully questioned and examined about tuberculosis. Particular attention should be paid to the development of tuberculosis in the patient group receiving infliximab.
